# Domestication of farmed fish *via* the attenuation of stress responses mediated by the hypothalamus–pituitary–inter-renal endocrine axis

**DOI:** 10.3389/fendo.2022.923475

**Published:** 2022-07-22

**Authors:** Yao Lu, Chuang Shi, Xia Jin, Jiangyan He, Zhan Yin

**Affiliations:** ^1^ State Key Laboratory of Freshwater Ecology and Biotechnology, Institute of Hydrobiology, Chinese Academy of Sciences, Wuhan, China; ^2^ The Innovative Academy of Seed Design, Chinese Academy of Sciences, Beijing, China; ^3^ Hubei Hongshan Laboratory, Wuhan, China; ^4^ Hainan Yazhou Bay Seed Laboratory, Sanya, China

**Keywords:** farmed fish, HPI signaling, domestication, genetic breeding, stress response

## Abstract

Human-directed domestication of terrestrial animals traditionally requires thousands of years for breeding. The most prominent behavioral features of domesticated animals include reduced aggression and enhanced tameness relative to their wild forebears, and such behaviors improve the social tolerance of domestic animals toward both humans and crowds of their own species. These behavioral responses are primarily mediated by the hypothalamic–pituitary–adrenal (inter-renal in fish) (HPA/I) endocrine axis, which is involved in the rapid conversion of neuronal-derived perceptual information into hormonal signals. Over recent decades, growing evidence implicating the attenuation of the HPA/I axis during the domestication of animals have been identified through comprehensive genomic analyses of the paleogenomic datasets of wild progenitors and their domestic congeners. Compared with that of terrestrial animals, domestication of most farmed fish species remains at early stages. The present review focuses on the application of HPI signaling attenuation to accelerate the domestication and genetic breeding of farmed fish. We anticipate that deeper understanding of HPI signaling and its implementation in the domestication of farmed fish will benefit genetic breeding to meet the global demands of the aquaculture industry.

## Introduction

Domestication is the process of adapting wild organisms for human use. Most domestic animals are raised by humans for food. Typically, this process extends over generations and involves developmental effects within each generation, leading to the gradual culmination of genetic changes across generations (1–3). Nearly 10 thousand years ago, some animals have been tamed for food or hunting. Goat is one of the first domesticated species, following the long-term selection of animals and breeds carrying specific traits related to meat, fur, milk, and docile behavior (2, 4). Thus, animals are artificially selected for traits preferred by humans, including productivity, fecundity, and tameness in captivity to reduce sensitivity to the stress caused by crowding, restricted movement, and parasitism as well as changes in the environment, predation, and food sources (2, 3). Compared with their wild ancestors, domesticated animals evolve distinct morphological, physiological, and behavioral traits during adaptation. Specifically, reduced aggression and enhanced tameness are the most prominent physiological and behavioral features of all domesticated animals (3, 5, 6). Another most evident change is the loss of fear of humans. These behavioral changes involved in increasing the threshold of within- and between-species aggression are perhaps equally important (7, 8). Meanwhile, while living under artificially controlled environments with abundant care and food, anxiety for avoiding predators and agility to search for food, which are behaviors mediated by neuroendocrine responses, are no longer essential for the survival of domestic animals. Moreover, reduced fear responses could also be associated with the decreased levels of the basal metabolism of domestic animals, thus improving their feed conversion efficiency (FCE) and further benefiting animal husbandry (9).

The hypothalamus–pituitary–adrenal (inter-renal in fish) (HPA/I) endocrine axis is the major neuroendocrine system responsible for the regulation of stress responses in animals (10, 11). This axis constitutes a complex set of positive and negative feedback loops between the neuro-endocrine system and peripheral tissues and organs, mediating adaptive physiological responses to stress. This complex system involves the brain (mainly hypothalamus) and a master endocrine gland (i.e., pituitary) as well as various hormones, CRH (corticotropin-releasing hormone, CRH) and their receptors (12). Upon the activation of the sympathetic nervous system, those hormones and neuropeptides are synthesized and released. The released hormones bind their corresponding receptors, such as the CRH receptor (CRHR) for CRH on the pituitary cells to promote the production of pro-opiomelanocortin (POMC) peptides. The POMC peptides are the pituitary precursors of circulating melanocyte-stimulating hormones (MSHs), adrenocorticotropin hormone (ACTH), and β-endorphin (13, 14). MSHs stimulate the rapid translocation of melanosomes (melanin granules) in dermal melanophores, leading to color change, which occurs in response to environmental changes to match the surroundings, typically for camouflage to either avoid predation or aid in foraging. In addition to their stimulatory effects on pigment cells, MSHs can also suppress appetite by acting on receptors in the hypothalamus of the brain (15–17). Upon cleavage from the POMC peptide, another pituitary peptide ACTH binds the melanocortin 2 receptor (MC2R) in the adrenal gland to stimulate cortisol synthesis and release. Cortisol is commonly referred to as the “stress hormone”, because its levels are usually elevated under stressful conditions. Cortisol affects the heart rate, breathing pattern, and other aspects of the body’s “fight or flight” responses (18, 19). Cortisol receptors are ubiquitous in the body and mediate diverse functions, including the maintenance of blood sugar levels, protection against stress, and suppression of inflammation (20, 21).

Domestication of farmed fish is also the process of artificial selection for traits preferred by humans, such as productivity, fecundity, and tameness (22, 23). Based on the comparative analyses on the compositions involved in the HPA/I signaling cascades between mammals and fish, the present review focuses on tameness achieved *via* the attenuation of stress responses mediated by the HPA/I endocrine axis through the selection of genetic variants in animals during domestication. Specifically, we describe how artificial selections shape tameness and simultaneously improves related productivity traits, such as food intake, body weight gain, and FCE. The association between stress response and reproduction has been extensively reviewed elsewhere (24, 25).

## HPA/I axis signaling cascade

### CRH and related peptide family

CRH is a peptide released in the hypothalamic region of the brain, which primarily known for its role in the control of the stress response in vertebrates (26). Over the last 30 years, many CRH paralogs with similar structures, including sauvagine (amphibians), urocortin (urotensin-1 in fish), urocortin-2 (UCN-2), and UCN-3, have been identified (27). Through phylogenetic analyses, it has been suggested that the CRH-related peptide family belongs to the diverse assemblage of secretin- and calcitonin-based peptides. These related peptides also include pituitary adenylate cyclase-activating peptide (PACAP), vasoactive intestinal peptide (VIP), glucagon, and teneurin C-terminal associated peptide (TCAP). In phylogenetic analyses of these peptides, each family formed a distinct clade: CRH, neuropeptide Y, secretin, TCAP, and calcitonin (28). Among these, CRH sequences are relatively conserved within the UCN/sauvagine or UCN2/3 lineages, indicating that the peptides in this clade may be derived from the CRH peptide family in chordates.

In vertebrates, the CRF and UCN peptides act to integrate the sensory systems and behavioral and homeostatic responses for the primary control of the HPA/I axis. They confer behaviors resembling the physiological response to stress and environmental changes. In *Crh*-deficient mouse models, little effect on the basal release of ACTH and prohormone convertase-2 (PC2), while markedly impaired adrenal responses to various acute stressors, such as restraint and fasting, have been observed. Thus, CRH is essential for ACTH secretion under stress, and it affects the release of prohormone convertase-1/3 (PC1/3) in the anterior pituitary. (29). Similar to mammals, CRH is principle hypothalamic initiator of the stress response in fish (30). Due to the teleost-specific whole-genome duplication, two *crh* homologs named *crha* and *crhb* were identified in teleost (31, 32). The expression of *crha* has been mainly observed in the retina, with weak expression in the brain in fish. By contrast, *crhb* is mainly expressed in the central nervous system and the caudal neurosecretory system. Therefore, it is generally believed that CRHb was mainly related to the control of ACTH secretion in the pituitary gland, which have been proved in many fish species, such as common carp, goldfish and rainbow trout (33–36). The effects of CRH on stress response have been investigated in fish. Central administration of CRH increased rhythmic locomotion in chinook salmon (37). Furthermore, downregulation of dark-stimulated melanosome dispersal and camouflage behavior were observed in *crhb* knockdown zebrafish (15). Although *crha*- or *crhb*-deficient zebrafish models have been generated previously, no information on their phenotypes has been reported (38). In teleost, the expression of *crha* and *crhb* are also found in peripheral tissues, such as head kidney, while the roles of CRHs in peripheral tissues remains unknown (39).

Two *crh* receptor genes *crhr1* and *crhr2* have been identified in most vertebrates, which belong to the class II seven-transmembrane G-protein-coupled receptor superfamily of receptors (GPCRs). Of these, CRHR1 is the dominant receptor in the HPA axis that mediates the anxiety response of the limbic system in mammals (40). In addition, CRHR1 mediates motor activation and anorexia, while CRHR2 is important for CRH-induced appetite suppression. *Crhr2*-deficient mice were hypersensitive to stress and exhibited anxiogenic behaviors (41). Furthermore, CRHR2 plays pivotal roles in the regulation of energy expenditure and response to homeostatic challenge (42). Intriguingly, the extent of impairment of the HPA axis response to stress was greater in mice lacking both *Crhr1* and *Crhr2* than in those lacking *Crhr1* alone, indicating that both *Crhr1* and *Crhr2* are critical for homeostasis during physiological responses to stress (43).

Two forms of CRH receptors were identified in fish. Although in tetrapods, it has been reported that CRH has higher affinity for CRHR1, in teleosts both CRH ligands have similar affinity for both CRH receptors (44). Depletion of *crhr1* in zebrafish did not increase cortisol levels and exhibited normal activity under acute stress (45). *Crhr2* deficiency was also generated in zebrafish without much phenotype description (46). *Crhr1* and *crhr2* gene knockouts were also performed in medaka. Upon exposure to high temperature stress, biallelic mutants of both loci (*crhr1* and *crhr2*) did not undergo female-to-male sex reversal which was observed in wild type fish (36). Furthermore, it has been observed that the attenuated HPI axis and reduced stress response to novel tank test can be seen with a genomic polymorphisms in *crhr2* gene locus in gibel carp (9). Major phenotypes in *crh*-related genes knockout or knockdown fish model (zebrafish or medaka) were summarized and compared with those of wildtype siblings in **Table 1**. These studies with the related genetic models indicate that the conserved functions of CRH and CRHR in HPA or HPI axis signaling existed in vertebrate models.

**Table 1:** Phenotypes of CRH-related gene knockout or knockdown mouse and fish models.

Gene	Major phenotypes in mouse model	Major phenotypes in fish model
*Crh or crhb*	Decreased glucocorticoid action; Reduced plasma ACTH levels; Reduced response to pain stress. (29, 47).	Decreased melanosome dispersal under dark-light stimulation. (15).
*Crhr1*	Atrophied adrenal gland medulla; Decreased glucocorticoid secretion; Reduced ACTH levels induced by stress; Increased exploratory activity; Reduced anxiety-related behavior. (40)	Failed to elicit a cortisol response to stress; Inhibited stress-induced hyperactivity; Decreased sex reversal rate under high temperature. (36, 45).
*Crhr2*	Hypersensitivity to stress; Increased anxiety-like behavior; Increased stress-induced glucocorticoid levels; Lower body fat but higher food intake on high-fat diet (42, 48)	Decreased sex reversal rate under high temperature. (36).
*Crhr1* and *Crhr2*	Normal basal ACTH levels; Reduced anxiety-related behavior in female; Exhibited more anxiogenic in male. (49)	No sex reversal under high temperature stress (36).

### POMC and its derived peptides

Mouse POMC is the polypeptide precursor of multiple hormones and neuropeptides, including MSHs (α-MSH, β-MSH and γ-MSH), ACTH, and β-endorphin. The *pomc* gene is predominantly expressed in the pituitary gland (50). Although its transcripts have been detected in many other tissues such as ovary and thyroid, no evident translation assays have been reported. These peripheral tissues express the *pomc* gene as an 800-base mRNA that lacks the 5’ end of the 1200-base pituitary transcript. The missing region encodes the signal peptide sequence, and thus, it is unlikely that any translation product would be secreted. Hence, no obvious phenotypes related to these additional tissues have been detected (50–52). Therefore, the functional significance of this hormone in tissues other than the hypothalamus and pituitary may be negligible. The active forms of MSHs, ACTH, and β-endorphin are encoded by the *pomc* gene. In a mouse model lacking *Pomc*, ACTH deficiency, impaired pigmentation, enhanced food intake, and obesity were observed. Observations in majority of the *Pomc* mutant mouse models revealed simultaneous dysfunction of active POMC*-*derived peptides (53–55). However, a relatively mild phenotype of the lack of opioid analgesia was noted in a mutant mouse model developed through the targeted deletion of β-endorphin at the C terminus of *Pomc* (56). Due to genome duplication, two *pomc* paralogs named *pomca* and *pomcb* were identified in zebrafish (57, 58). Compared with POMCa, POMCb lacks N-terminal sequences, only α-MSH and β-endorphin sequence were retained. Furthermore, POMCb-derived β-endorphin lost the opioid core sequence YGGF (58). Therefore, zebrafish *pomcb* was regarded as a pseudogene occasionally (15). Thus, only *pomca* was expressed in pituitary gland and might be responsible for the HPI axis signaling (59).

POMC polypeptides are cleaved by pro-hormone convertases (PCs) at specific amino acid sequences *in vivo*. In humans and other mammalian models, the types of PCs and their processing in specific tissues determine the production of certain active peptides. For instance, PC1/3 has been implicated in the processing and production of ACTH and β-endorphin in the anterior pituitary, while PC2 controls the processing and production N-POMC, ACTH, and MSHs in the pars intermedia of the pituitary (50). Therefore, the specific functions of certain hormones may be explored in mutant animal models lacking a particular type of PCs. For instance, obesity and altered ACTH levels were detected in human patients lacking PC1/3 (60), although no obvious defects were observed in PC1/3-deficient mice (61). In human patients and mouse models lacking PC2, defects related to fat metabolism and insulin signaling were observed (61, 62).

Five melanocortin receptors (MC1R–MC5R) have been identified in vertebrates. These receptors can be differentiated based on their tissue distribution and ligand affinity (**Table 2**). Loss-of-function mutations of *Mc1r* often lead to the development of a paler phenotype in mammal. Meanwhile, the constitutive activation of *Mc1r* induced by mutations is typically associated with the development of a darker phenotype (63–65). Furthermore, the loss-of-function mutations of *mc1r* also impair pigmentation patterns in zebrafish (66). Therefore, MC1R primarily mediates MSH signaling for the pigmentation. Since MC2R is a specific receptor for the ACTH derived from the POMC polypeptide, ACTH is involved in adrenal development and corticosterone production. While depletion of *Mc2r* reduced rapid locomotor response to acute stressors as expected, the neonatal lethality as a result of hypoglycemia observed in three-quarters of the *Mc2r* mutant mice has been reported. (67, 68). Although *mc2r* mutant zebrafish also exhibited decreased rapid locomotor response to acute stressors, the neonatal lethality was not observed (68). MC3R and MC4R were considered to regulate energy homeostasis in vertebrates. *Mc3r* -deficient mice exhibited increased fat mass, reduced lean mass, and enhanced FCE compared with their wildtype control siblings (69). In addition, MC3R is a critical regulator for melanocortin signaling and rheostatic control on energy storage in mammal (70). Although *Mc3r* -deficient mice were neither hyperphagic nor did they develop diabetes under normal diet, *Mc3r* mutant mice lost significantly more of their body weight under caloric restriction and gained weight at a much faster rate after energy-rich chow (70, 71). In *Mc4r* -null mice, the maturity-onset obesity syndrome associated with hyperphagia, hyperinsulinemia, and hyperglycemia have been observed, and these mice exhibited increased linear growth (72). Furthermore, mice lacking both MC3R and MC4R exhibited even more significant body weight gain than those lacking MC4R alone. Therefore, both MC3R and MC4R serve non-redundant roles in POMC signaling for the regulation of energy homeostasis in mice (69). Natural coding mutations cause increase in appetite and starvation resistance of cavefish compared with control fish (73). However, reduced food intake and the same size were observed in *mc3r* or *mc4r* -deficient zebrafish compared with those of the wild type fish. Double knockout of *mc3r* and *mc4r* in zebrafish shown the same results. It seems that there is a compensatory mechanism that overrides the effect of genetic manipulations of the melanocortin system in zebrafish (74). Targeted disruption of mice *Mc5r* impeded the functions of multiple exocrine glands, and the mutants exhibited severe defects in water repulsion and thermoregulation, indicating that ACTH/MSHs signaling coordinates the functions of exocrine glands *via* MC5R (75). *Mc5r* transcript was found in brain, pituitary, kidney, and skin in fish (35). However, the studies investigated the roles of MC5Rs in fish are inadequate. No loss of function or mutant *mc5r* fish were reported. The typical phenotypes of melanocortin receptors gene mutation mouse and fish models are summarized in the **Table 3**.

**Table 2:** Melanocortin receptors and their ligand selectivity.

Melanocortin receptor	POMC-derived peptides	Tissue distribution
MC1R	α-MSH=ACTH>β-MSH>γ-MSH	Melanocytes of the skin and hair follicles
MC2R	ACTH only	Adrenal cortex
MC3R	α-MSH=ACTH=β-MSH=γ-MSH	Hypothalamic and limbic regions of the brain
MC4R	α-MSH=ACTH>β-MSH>γ-MSH	PVN of the hypothalamus, central nervous system
MC5R	α-MSH>ACTH>β-MSH>δ-MSH	Embryo, exocrine glands, peripheral tissues

MSH, melanocyte-stimulating hormone; ACTH, adrenocorticotropic hormone; PVN, paraventricular nucleus; POMC, pro-opiomelanocortin.

**Table 3:** Comparison the major phenotypes of melanocortin receptors gene mutation in mouse and zebrafish models.

Gene	Major defects observed in mouse model	Major defects observed in zebrafish model
*Mc1r*	Abnormal eumelanin synthesized; Yellow coat color. (76)	Reduced countershading; Exhibited general paling. (66)
*Mc2r*	Elevated ACTH levels; Undetectable corticosterone levels; Reduced stress response; High neonatal lethality and hypoglycemia; Adrenal gland hypoplasia. (77)	**Decreased interrenal steroidogenic genes expression;** **Reduced locomotor activity response to acute stress. (** 78 **;** 68 **)**
*Mc3r*	Normal body weight; Increased adiposity; Reduced lean mass. (69)	Retarded growth and; Reduced food intake; Decreased feeding rate; Normal body size at adult stage. (74)
*Mc4r*	Maturity-onset obesity syndrome; Increased adiposity and lean mass; Increased body weight and body length; Hyperphagia, hyperinsulinemia, and hyperglycemia. (72, 79)	Reduced food intake; Invariable body size. (74)
*Mc3r* and *Mc4r*	Increased body weight compared with that of the either single mutant. (69)	Reduced food intake; Invariable body size; Much more lower feeding rate. (74)
*Mc5r*	Defective thermoregulation; Reduced water repulsion; Normal appetite and body weight. (75)	

The vital roles on diverse physiological systems of POMC-derived peptides have been intensively studied. Although as an archetypal polypeptide precursor, understanding the precise processing steps for the cleavage of POMC peptides in each specific tissue remains challenging. Overall, the processing of POMC peptides expressed in the hypothalamic neurons affect the regulation of appetite, energy homeostasis, and body composition. In the pituitary, correct processing of POMC peptides is essential to maintain HPA/I signaling for regulating several aspects of stress response.

### Hormones of the adrenal (interrenal) gland

Adrenal glands are small glands located on top of both kidneys in mammals. These glands are composed of two parts, namely the cortex and medulla. The adrenal cortex is the outer region, which is divided into three separate zones: zona glomerulosa (ZG), zona fasciculate (ZF), and zona reticularis (ZR) (80). The adrenal glands release specific hormones directly into the bloodstream. While many of these hormones are related to the body’s response to stress, others are vital for survival. The biosynthesis of many of the adrenal hormones is regulated by ACTH produced in the anterior pituitary and its receptor MC2R (81). Among these, the most important hormones are glucocorticoids (cortisol in humans and cattle and corticosterone in most rodents), mineralocorticoids (aldosterone), and androgen precursors (82). These hormones are essential for homeostasis, development, and survival. The common endocrinopathies caused by adrenal gland dysfunction include Cushing’s syndrome, Addison’s disease, hyperaldosteronism, and congenital adrenal hyperplasia (CAH) (80). Several key enzymes are involved in the synthesis of steroidal hormones in the adrenal cortex. Specifically, cytochrome P450 family 11 subfamily B member 2 (CYP11B2) and hydroxylase activity unit of cytochrome P450 family 17 subfamily A member 1 (CYP17A1) are involved in the synthesis of mineralocorticoids exclusively in ZG, whereas CYP11B1 is involved in the synthesis of glucocorticoids in ZF and ZR. The lyase activity unit of CYP17A1 is essential for the production of androgen precursors dehydroepiandrosterone (DHEA), DHEA-sulfate (DHEAS), and androstenedione. Under normal physiological conditions, the response of adrenocortical cells to ACTH occurs in two phases: an acute phase, lasting seconds to minutes, and a more sustained phase (80, 83). 

It is worth noting that fish do not have a discrete adrenal gland with distinct cortex and medulla (84). The adrenal cortex homolog in teleost is called the interrenal, because together with chromaffin cells (counterpart of adrenal medulla) and embedded inside the anterior part of the kidney, commonly referred to as the head kidney (85). Although there are differences in structure and ontogeny between fish interrenal and mammalian adrenal cortex, both of them can produce steroid hormones with similar functional entities (86).

Fundamentally, adrenocortical synthesis is the process of steroidogenesis, which involves the mobilization and delivery of free cholesterol from lipid droplets to the inner mitochondrial membrane and then to endoplasmic reticulum as well as every catalytic reaction in the synthesis of cholesterol to aldosterone, cortisol, and DHEA/DHEAS. Many enzymes belonging to the cytochrome P450 (CYP) and hydroxylsteroid dehydrogenase (HSD) families are involved in adrenal steroidogenesis, including steroidogenesis in the gonads. Additionally, steroidogenic acute regulatory protein (StAR) and nuclear receptor NR5A/steroidogenic factor 1 (SF1) are involved in adrenal steroidogenesis. All pathways implicated in the adrenal cortex and gonads are closely interconnected. Although some defects caused by adrenal gland disorders are linked to mutations in *StAR*, *HSD3B2*, *CYP21A2*, and *CYP17A1* in human patients and animal models, these models generally exhibited adrenocortical insufficiency and gonadal abnormalities. Thus, the precise function of each adrenal steroidal hormones could not be clarified based on analyses using these models (81, 87).

The effects of cortisol are mediated through intracellular receptors, glucocorticoid receptor (GR, encoded by the *nr3c1* gene) and mineralocorticoid receptor (MR, encoded by the *nr3c2* gene) (88). In mammals, GR was a specific receptor for cortisol and typically expressed ubiquitously. Whereas MR can be activated by aldosterone and cortisol, which show similar binding affinity (89). In contrast, it has generally been thought that there is no aldosterone synthesis in teleost fish. Both of GR and MR are specific receptor for cortisol in teleost (90). Owing to the much higher levels of circulating and intracellular glucocorticoids, MR primarily acts as GR. Except in the aldosterone target cells, 11β-hydroxysteroid dehydrogenase type II (11β-HSD2) efficiently metabolizes intracellular cortisol or corticosterone, specifically activating MR–aldosterone signaling (91).

Glucocorticoids were originally named as such based on their ability to promote gluconeogenesis in hepatocytes (92). As a ubiquitously expressed nuclear receptor, GR mediates the action of glucocorticoids in various tissues. However, completed depletion of GR lead to neonatal lethality in mice due to respiratory failure caused by impaired lung development (93). Moreover, mice completely lacking MR died within 1–2 weeks postnatally due to renal salt wasting and hyperkaliemia, with elevated plasma renin and aldosterone levels (94). Meanwhile, conditional targeted deletion of GR or MR in various cells provided opportunities to characterize this receptor and its effects on stress response *in vivo* (95, 96). Unlike mouse models, Loss of GR or MR functions in zebrafish models exhibited normal survival rate and live to adult stage. Depletion of GR reduce stress response, improved muscle glucose availability and increased somatic growth in zebrafish (97–99). Depletion of MR exhibited social behavioral deficit, more active and slept less at night compared with wild type fish, but no significant different of cortisol levels, body size and rapid locomotor response to acute stressors were observed between two groups (68, 100, 101).

The mammalian adrenal gland comprises two endocrine tissues with embryological origin: steroid-producing adrenal cortex derived from the mesodermal populations and catecholamine-producing chromaffin cells in the adrenal medulla derived from the neural crest precursor cells. Epinephrine and norepinephrine are the major secretory products of adrenomedullary chromaffin cells, in addition to some other transmitters, neuropeptides, and proteins. The released catecholamines bind the α- and β-adrenergic receptors to promote respiration, blood pressure, and heart rate as well as some catabolic activities. Tyrosine hydroxylase (TH) and phenylethanolamine N-methyltransferase (PNMT) are the two key enzymes involved in epinephrine biosynthesis from tyrosine in the adrenal medulla. The expression of *Th* and *Pnmt* is regulated by CRH from the hypothalamus and glucocorticoids from the adrenal cortex, respectively (102, 103). In addition, the stability of the PNMT protein is regulated by glucocorticoids from the adrenal cortex (104). Epinephrine production in the adrenal medulla is induced by neural stimuli through the splanchnic nerve innervation of the gland. Meanwhile, in response to splanchnic nerve stimulation, the secretory products of the adrenal medulla can also affect adrenocortical function by regulating steroidogenesis (105). Taken together, the function of the adrenal gland, as an endocrine organ bearing tissues with two embryologically distinct origins, and its responses to stress are synchronously regulated by HPA signaling and splanchnic innervation.

Overall, at the systemic level, stressors can activate physiological responses *via* the sympathetic branch of the autonomic nervous system, followed by the activation of the HPA axis and rapid responses mediated through splanchnic nerve activation, triggering catecholamine release. The body’s stress response can generally be described as fight or flight, induced either directly *via* hormone release mediated by the HPA axis or indirectly *via* splanchnic nerve activation.

## Domestication of wild fish for farming *via* attenuated HPI axis signaling

### History of domestication in fish

Typically, domestication of animals accompanied by the advancement of civilization offers humans many benefits in their daily life, including usage as farm animals, food sources (meat, milk, and eggs), or pets (social companions and/or protection) (106). The first experiments in the domestication of terrestrial animals arose independently around 12000 years ago in various areas over the world (107, 108). In the past, it was generally thought that the first trial of farmed fish domestication is much later than those in terrestrial animals, according to the evidence that found on art from Egyptian tombs suggests management of Nile tilapia (*Oreochromis niloticus*) by 1500 BC (109, 110). However, another scientific research found that the first trials of farming fish species for human consumption might date back to 8,000 years ago, with the managed aquaculture of common carp (*Cyprinus carpio*) at the Early Neolithic Jiahu site of Henan Province, China (111). This discovery greatly pushes forward the history of fish farming.

At about 460 BC, the oldest written work, Yang Yu Jing (Fish Culture Guidebook), on aquaculture was completed (112). Few centuries later, the farming of three other carps started in China: the silver carp (Hypophthalmichthys molitrix), the bighead carp (Hypophthalmichthys nobilis) and the grass carp (Ctenopharyngodon idella) (113). Thereafter, the history of farmed fish varies enormously in different regions. In Europe, the farming of common carp in ponds was already developed during the Middle Ages (110). The Italian “Vallicoltura” (extensive farming of various marine species in coastal lagoons and large open waterbodies) dates back to the 15th century (114). The French trout culture started around the second half of the nineteenth century (110). In North America, aquaculture started about 100 years ago (115). In Africa, aquaculture dates back to the 1940s. In Australia, New Zealand, and diverse Pacific Island states, the development of aquaculture is even more recent (110, 116).

Goldfish are the first domesticated ornamental fish, shaped by purposefully or biased selection from approximate 1800 years ago in China and have now developed more than 300 varieties or strains (117). Over the past four decades, aquaculture became the fastest-growing food production sector in the world. Now, over half of annually harvested fish weight destined to human consumption are farmed globally (118, 119). To better describe the diverse strategies for fish production, Fabrice Teletchea and Pascal Fontaine proposed a new classification comprising five levels of ‘domestication’ with 1 as the least to 5 as the most domesticated. Among the 313 fish species listed in the FAO (Food and Agriculture Organization) in 2009, only 30 fish species have reached level 5 (120). Moreover, despite impressive expansion in aquaculture over the past four decades, only minority fish species, such as common carp, silver carp and Nile tilapia et.al., which have been well domesticated for aquaculture production (121). Therefore, although the farming fish is a traditional aquaculture practice, particularly in Asia, the domestication of most farmed fish remains at early stages, and fingerlings of many farmed fishes are obtained from the wild even today. To continue current exponential growth of aquaculture, which is heavily relied fingerlings from wild population, studies and improvements on domestication of these fish species are required.

### “Domestication syndrome” in animals: from mammals to fish

Among the traits required in all domestic animal, the core set of characteristics are increased docility and tameness (122, 123). However, in addition to these, coat color changes as well as morphological changes in teeth and craniofacial features are common during domestication. The typical set of traits in domesticated mammals is called the “domestication syndrome” (124). In most cases, wild animals must adapt to artificial environments and captivity during gradual transformation over many generations (125). During this course, the major difference between the wild and domestication animals was their living environment, including predation pressure, weather, and food availability. Thus, the traits of agility and sensitivity of the targeted animals, which were essential for survival in the wild, would be attenuated in controlled conditions, and beneficial traits, such as productivity and fecundity, would be further selected (126).

Fish domestication is a continuous process that improves production traits for aquaculture and human consumption (117, 127). Therefore, improving the growth traits of farmed fish is acknowledged as the primary goal in the process domestication, which resulted in excellent growth traits exhibited in most of all the domesticated fish, Interestingly, compared with wild type ancestors, these domesticated fish commonly exhibited reduced stress response and cortisol levels which were belonged to core set of “domestication syndromes” in mammals. These indicate that the “domestication syndromes” is also exist in fish and there is an internal connection between growth traits and tameness in fish.

### Changes of HPI axis during domestication in fish

Increased evidences have revealed that the longer fish are domesticated, the lower their levels of cortisol relative to wild fish after exposure to acute or chronic stressors (128). The stress response of teleost fish is highly similar to that of terrestrial vertebrates, which mediated *via* the brain–sympathetic–chromaffin cell axis (equivalent of the brain–sympathetic–adrenal medulla axis) and the HPI axis (equivalent of the HPA axis). In fish, along with CRH and POMC-derived peptides, high circulating levels of catecholamine as well as cortisol are essential for the restoration of hydromineral homeostasis in response to external stressors (88). Two neuronal populations are present in the hypothalamic region of fish, including neurons co-expressing neuropeptide Y (*npy*) and agouti-related protein (*agrp*) and those co-expressing *pomc* and cocaine- and amphetamine-regulated transcript (*cart*). The presence of these stress responsive neurons in fish indicates that the regulatory pathways for energy homeostasis *via* the HPA/I axis have been conserved through the vertebrate evolution (129).

However, most studies exploring the endocrine effects of hormones involved in HPI signaling were performed by assessments through the administration of exogenous hormones or their analogs. In experiments involving exogenous administration, the applied hormones may not necessarily mimic the physiological actions of the actual hormones *in vivo*. In recent years, more credible studies have been performed using genetic fish models generated through advanced gene editing technique available recently with the clustered regularly interspaced short palindromic repeats/Cas 9 (CRISPR/Cas9) system. Zebrafish lacking glucocorticoid receptor (GR) exhibited high levels of whole-body cortisol, which were associated the upregulation of *crh* and *pomca* transcripts along the HPI axis. In addition, reduced exploratory behavior and increased fat deposition were observed in GR-deficient zebrafish (97, 130). Enhanced somatic growth together with inhibited food intake, promoted protein synthesis and attenuated protein catabolism were observed in GR-null zebrafish. Significant less protein breakdown after 7 days fasting in GR-null zebrafish compared with those of wildtype zebrafish has been observed (98). Intriguingly, the stress-induced elevation of plasma glucose levels was abolished in GR-null zebrafish, which was associated with enhanced glucose uptake in the muscle tissues of the mutants. Therefore, GR-mediated signaling is responsible for the elevation of plasma glucose and inhibition of glucose uptake in peripheral tissues (98). Furthermore, the loss-of-function mutations in MC1R impaired pigmentation patterns in zebrafish (66). Based on observations in zebrafish mutants of MC2R, GR, and MR, locomotor responses to certain environmental stresses require actions mediated by MC2R and GR but not MR (68). Although MC3R- or MC4R-deficient zebrafish models exhibited normal body size, reduced food intake was observed and thus elevated FCE indirectly (74).

**Figure 1:**
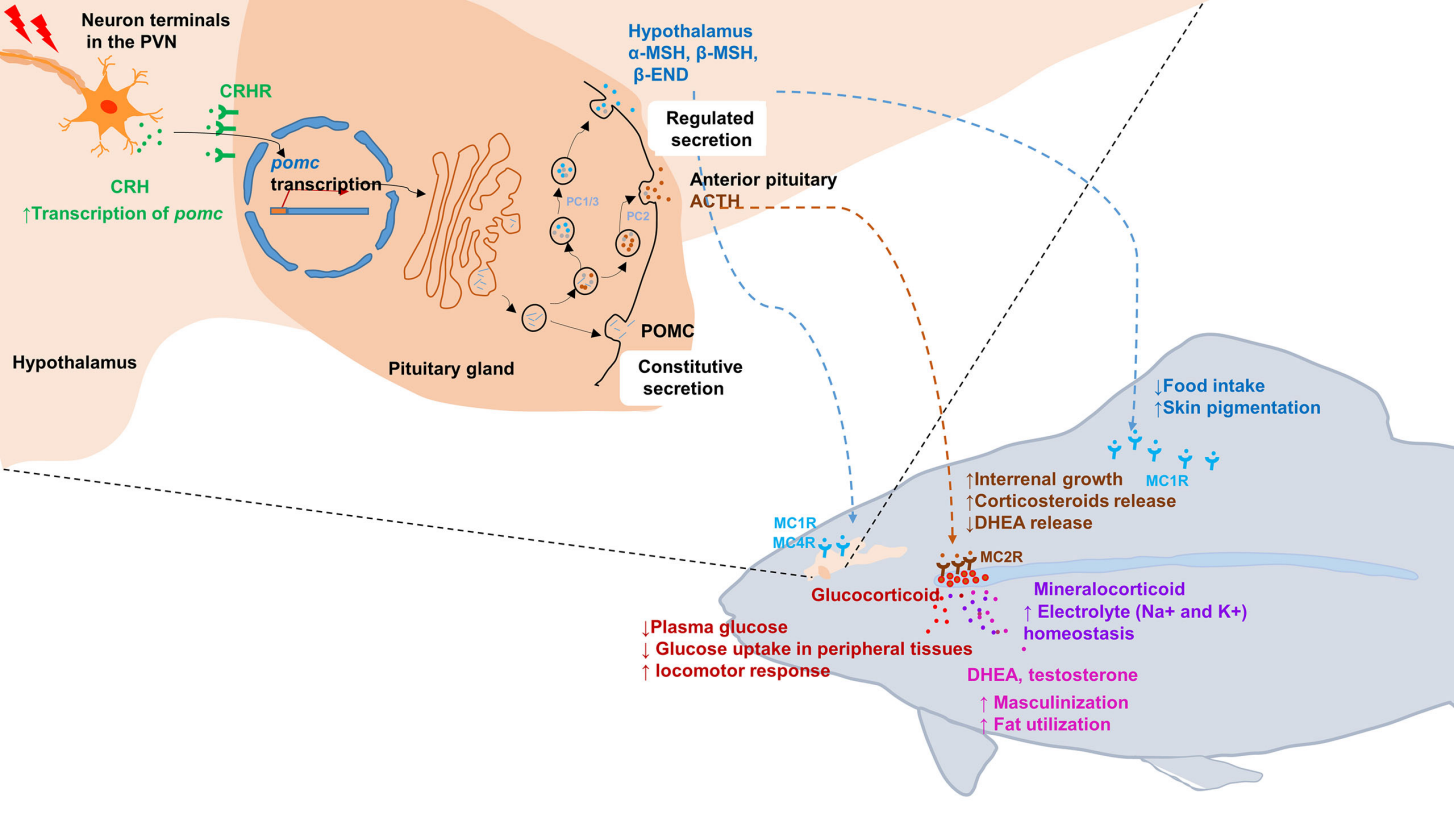
Schematic illustration of the hypothalamic–pituitary–inter-renal endocrine axis in stress and metabolisms in fish. Top left portion shows synthesis of POMCa in the hypothalamus and pituitary gland. In response to stress, CRH in the PVN is released into pituitary portal vessels that reach the anterior pituitary gland. Binding of CRH to its receptor CRHR on pituitary corticotropes induces the transcription of *pomc*. After translated into pro-hormones, the POMC polypeptides is cleaved into mature hormone by PC1/3 and PC2, including α-MSH, β-MSH, ACTH and β-END etc. Lower right portion indicates that the POMC-derived peptides bind with its corresponding receptors and activated downstream signaling. MSHs bind with MC1R to suppress appetite by increasing satiety in hypothalamus and promoting melanosomes dispersion in skin. Moreover, MSHs bind with MC4R to regulate energy homeostasis in hypothalamus. In fish interrenal gland, ACTH can specifically bind with MC2R to promote interrenal growth and syntheses and releasing of steroid hormones, including glucocorticoids, mineralocorticoid, DHEA and testosterone. Once these steroid hormones act on target tissues and organs, a series of physiological and metabolic activities are initiated, such as glucose uptake, electrolyte homeostasis, sexual differentiation and lipid utilization etc. ACTH, adrenocorticotrophic hormone; β-END, β-endorphin; CRH, corticotropin-releasing hormone; DHEA, Dehydroepiandrosterone; MC4R, melanocortin 4 receptor; MSH, melanocyte-stimulating hormone; PC, prohormone convertase; POMC, pro-opiomelanorcortin; PVN, paraventricular nucleus.

**Figure 2:**
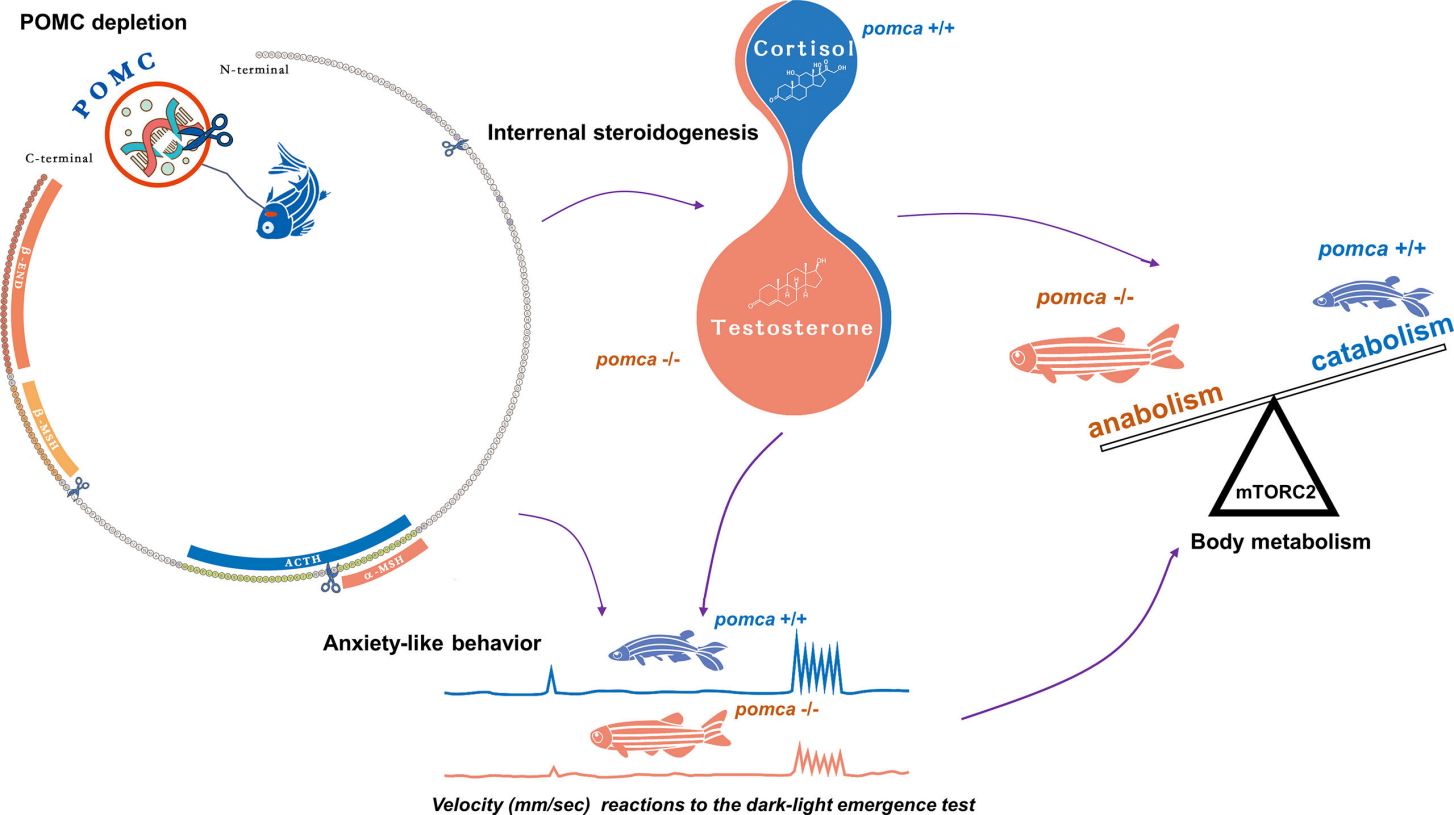
Schematic illustration of enhanced growth in *pomca* knockout zebrafish. Depletion of *pomca* disrupts interrenal steroidogenesis in zebrafish (59). Reduced plasma cortisol level and increased testosterone content are observed in *pomca* mutant zebrafish. Reduced cortisol level in POMCa-deficient zebrafish decreases oxygen consumption and inhibits anxiety-like behavior under dark-light emergence test. Furthermore, Hyperandrogenism is responsible for enhanced somatic growth without increasing adiposity in POMCa-deficient zebrafish. ACTH, adrenocorticotrophic hormone; β-END, β-endorphin; MSH, melanocyte-stimulating hormone; mTORC2, mammalian target of rapamycin complex 2; POMC, pro-opiomelanorcortin.

Aldosterone synthesis in the adrenal cortex is unique to terrestrial vertebrates and absent in ray-finned fish. In teleosts, aldosterone levels are extremely low and cortisol may serve as the regulator of GR and MR (131, 132). Subsequently, both progesterone and spironolactone were found to be the potential physiological ligands for MR in teleosts (133). Male Mozambique tilapia outgrow females. The expression of *pomc* in brain is female-biased and found to be mediated by estrogen. Therefore, the high estrogen level in females promotes the expression of *pomc*, which may lead to the slower growth of female tilapias (134). In zebrafish models, *pomca*-deficient zebrafish were generated in our laboratory recently. Both of these *pomca* mutant fish exhibited increased body weight (59, 134). Moreover, mutations in *pomca* in zebrafish impaired melanosome dispersal, increased food intake, suppressed anxiety-like behavior, and reduced basal metabolism. Additionally, *pomca* mutant zebrafish shown excessive muscle growth due to myocyte hyperplasia. Enhanced protein synthesis and fatty acid β-oxidation in muscle tissue were also observed in *pomca* mutant zebrafish compared with those of wild type zebrafish. These physiological processes resulted in enhanced body weight gain without obesity in the mutant zebrafish (59) (**Figure 1**). Additionally, based on the comparative phenotype analyses with the series of different mutations within zebrafish *pomca* locus generated in this laboratory, it has been found that not the food intake but cortisol levels and locomotion patterns were correlated with body weight in the *pomca*-deficient zebrafish models. These indicate that among the POMC-derived peptides, ACTH has been implicated as the key regulator of enhanced somatic growth observed in *pomca*-deficient fish. Interestingly, the phenotypes of hyperandrogenism and inducible masculinization have also been observed in *pomca*-deficient zebrafish mutants. It has been demonstrated that the hyperandrogenism is responsible for the non-obese phenotype observed in *pomca*-deficient fish (59) These reports suggest that the principal functions of the HPA/I axis are typically conserved across vertebrates. specific signals in fish differ from those in mammals in terms of certain aspects, such as the association of the hyperandrogenism in the *pomca*-deficient fish (**Figure 2**).

Nonetheless, from knowledge on the domestication of terrestrial animals, HPI axis-mediated stress responses of fish have been recognized in captive environments of aquaculture facilities. The negative correlation between fish somatic growth and cortisol levels has been studied. Low plasma cortisol levels associated with rapid growth have been reported in some farmed fish species, such as Eurasian perch (*Perca fluviatilis*), rainbow trout (*Oncorhynchus mykiss*), Atlantic salmon (*Salmo salar*), and arctic charr *(Salvelinus alpinus*), among others (135–138). High fecundity of fish can facilitate extensive collection of phenotypic records among close relatives of candidate strains for selection throughout the course of breeding. The reproductive biology of fish is suitable for the application of many genetics and breeding approaches. Recently, with the advancement of high-throughput sequencing technologies, several molecular markers and selection signatures have been identified through many gigabases of sequences of domesticated fish species (139–146).

However, unlike majority of the relatively old selection signatures in terrestrial animals, dating back hundreds of thousands of generations, genetic variations in traits of many farmed fish species have been subjected to selection only for a few tens of generations. The selection signatures of fish are confined to the small scale, due in part to the relatively recent domestication of fish and availability of limited resources, such as reference genomes and functional data. Only few genomic variations associated with the stress responses of farmed fish have been identified. Recently, selection sweeps of γ-aminobutyric acid receptor subunit gamma-2 (*gabrg2*) have been identified in farmed Atlantic salmon (145). Interestingly, *Gabrg2*-depletion promoted exploratory behavior in mice (147). Reduced fear and aggression have been observed in general transcription factor II-I repeat domain-containing protein 1(*Gtf2ird1*)-targeted mice (148). Moreover, the association of *gtf2ird1* with X has been reported in farmed Nile tilapia and coho salmon (146, 149). Among fish species, goldfish has been subjected to approximate 1800 years of domestication and selective breeding (112). Therefore, many domestication-associated selective sweeps affecting HPI signaling, including *agrp*, *pomcb-like*, *mc2r*, *mc4r*, and *mitfa*, among others, have been identified through genome-wide screening (144). In addition, a comparative analysis of persistent changes in the genomic DNA methylation patterns of wild and farmed European sea bass has revealed some epimutations, such as *grik4* and *gria4a*, in farmed European sea bass after 25 years of selective breeding (150). These results indicate the role for epimutations at the beginning of domestication, which may be exploited to fix certain genetic variants.

Within the mammalian ARC of the hypothalamus, POMC neurons are the anorexigenic neurons expressing POMC and CART. POMC neuronal activation is catabolic, which is critical for the maintenance of negative energy balance (151). Mutations in *pomc* result in hyperphagia and obesity. The transcription of leptin receptor (*lepr*) has also been detected in POMC neurons. Leptin is an adipocyte-derived satiety factor, and elevated *leptin* levels activate anorexigenic POMC neurons (152). Meanwhile, in mammals, a subset of POMC neurons expressing *lepr* is also essential to regulate the fasting-induced drop in *leptin* transcription and Leptin synthesis (153). However, no hyperphagia or obesity was observed in adult zebrafish lacking *lepr*, suggesting that the regulatory role of *leptin* in adipostasis is not conserved in vertebrates (154). Subsequently, however, some phenotypes of obesity and anxiety were reported in either *lepa*- or *lepb*-deficient zebrafish models (155, 156). Thus, POMC deficiency can promote body weight gain, impair coloration pattern, and alleviate anxiety-like behaviors in mice, humans, and zebrafish. The phenotype of obesity was observed in POMC-deficient human patients, mice, and Labrador Retriever dogs (53, 157, 158). However, this phenotype was not observed in *pomca*-deficient zebrafish. Moreover, transcript levels of *leptin* and *insulin* remained unchanged in *pomca*-deficient zebrafish (59). Therefore, majority of the stress responses mediated *via* the HPA/I axis are conserved across vertebrates, except for adipostasis, which may be related to the unique roles of *leptin* signaling in the control of obesity in zebrafish (154). Major phenotypes of mouse and zebrafish with POMC mutation were compared in **Table 4**.

**Table 4:** Comparison of POMC action in mouse and zebrafish model.

Phenotype	*Pomc* mutant mouse	*pomca* mutant zebrafish
Decreased stress response	Yes	Yes
Hyperphagia	Yes	Yes
Hypometabolism	Yes	Yes
Body weight gain	Yes	Yes
Hyperglucagonemia	Yes	No
Hyperinsulinemia	Yes	No
Obesity	Yes	No
Diabetes	Yes	No
Hyperandrogenism	No	Yes
Infertility	Yes	No
Partial lethal	Yes	No

Intriguingly, hyperandrogenism has not been reported in POMC-deficient mammals, further suggesting the presence of distinct mechanisms of leptin-POMC signaling and adrenal steroid synthesis in teleosts and mammals. Nevertheless, the phenotype of enhanced somatic growth without obesity caused by the attenuation of the HPA/I axis in teleosts would be well suitable for the domestication of farmed fish in aquaculture. Coincidentally, a set of polymorphisms at the *crhr2* locus was found to be associated with the enhanced FCE of the farmed allogynogenetic Gibel carp strain CAS III compared with that of the wild Gibel carp strain Dongting (DT) through 2b-restriction site-associated DNA (2b-RAD) sequencing and FCE correlation analyses. This set of polymorphisms downregulated *crhr2* transcript levels in the brain and pituitary tissues of the Gibel carp strain CAS III compared with those of the wild Gibel carp strain DT. Furthermore, defective HPI signaling was observed in the Gibel carp strain CAS III, such as decreased α-MSH protein levels, plasma cortisol concentration, and anxiety-like behaviors. Overall, these findings demonstrate that genetic variations associated with dysregulated HPI signaling, such as *crhr2* downregulation, are potentially useful in genetic selection for the improvement of growth and FCE of farmed fish, which would reduce fishmeal consumption and thereby indirectly facilitate sustainable fisheries (9).

## Conclusion and perspectives

Over the past four decades, selective breeding is one of most important methods in farmed fish breeding. However, it would take long period, such as Atlantic salmon which has accelerated its growth rate by 113% through five generations of selective breeding since 1975 (159). Furthermore, such a breeding strategy inadvertently resulted in germplasm degradation and brought great challenges to the aquaculture simultaneously (159, 160). Given the association between the attenuation of the HPA endocrine axis and domestication of terrestrial animals, it is reasonable to believe that “domestication syndromes” is also exist in fish and successful selective breeding of suitable farmed fish can be accelerated through the same concept.

Feed conversion efficiency (FCE) is an important characteristic of growth trait as well as growth rate. Despite the higher FCE compared with livestock species, the high cost of feed ranging from 30% to 70% of the total production budget is the primary hurdle of intensive fish farming. Therefore, improving FCE is a key to reduce production costs in aquaculture and to achieve sustainability for the aquaculture industry (161). Optimized feed formula is the primary strategy to improve FCE in the past. However, this approach has encountered the challenges in recent years, such as the shortage of fish meal or other raw materials. Only a limited amount of research has been devoted to use genetics, despite its potential (161). Genomic polymorphisms in gibel carp *crhr2* locus and knockout zebrafish *nr3c1* and *mc4r* could improve FCE in fish (9, 74). These studies suggest that attenuated HPI axis signaling using genetic approaches is a new strategy for FCE improving in farmed fish.

For future genetics and breeding of fish, meaningful genetic variants may be artificially selected *via* powerful genome-wide association studies, and the identified genetic polymorphisms, such as *crhb*, *crhr2*, *pomc*, *mc2r*, *mc4r* and *nr3c2* et al., may further be used as molecular markers. Subsequently, following whole-genome screening, genetic marker-assisted breeding and selection of farmed fish can be realized to effectively improve their tameness and productivity traits. In addition, as another potential tool for fish breeding with improved performance, gene editing targeting key molecules can be performed using highly precise and accurate methods to generate the genetic alterations, avoiding the wait for the occurrence of natural spontaneous mutations. Considering the rapidly growing global demand for high-quality animal proteins, accelerating the domestication of farmed fish would significantly mitigate the competition for water use.

Despite attenuated HPI axis signaling is a potential way for the acceleration of fish domestication, remaining questions need to be investigated in future: (1) Attenuated HPI axis signaling reduced fish stress response. What is the neuromodulation mechanism in it? (2) What is the molecular mechanism of cortisol in regulation of muscle tissue anabolism and catabolism in fish? (3) Although cortisol is the primary index for evaluation of stress response in fish, it is difficult to accurately determine plasma cortisol levels due to due to disturbance caused by procedure of anesthesia and out-of-water blood collection. Therefore, appropriate device needs to be developed for monitoring cortisol levels in fish. (4) Knocking out the genes in HPA axis, such as *pomc*, *mc2r* and *nr3c1*, caused neonatal lethality in mammals. Why no fatal defects were observed in the mutants of these genes in fish? (5) What are effects of attenuated HPI axis signaling on other economic traits in fish, such as hypoxia tolerance and disease resistance?

## Author Contributions

ZY, YL, and CS drafted and revised the paper. XJ and JH revised the paper. All authors contributed to the article and approved the submitted version.

## Funding

This work was supported by the National Key R&D Program (2018YFD0900404); Pilot Program A Project from the Chinese Academy of Sciences (XDA24010206); National Natural Science Foundation of China (32102783); State Key Laboratory of Freshwater Ecology and Biotechnology (2019FBZ05) and Hainan Yazhou Bay Seed Laboratory (B21Y10106).

## Conflict of Interest

The authors declare that the research was conducted in the absence of any commercial or financial relationships that could be construed as a potential conflict of interest.

## Publisher’s Note

All claims expressed in this article are solely those of the authors and do not necessarily represent those of their affiliated organizations, or those of the publisher, the editors and the reviewers. Any product that may be evaluated in this article, or claim that may be made by its manufacturer, is not guaranteed or endorsed by the publisher.
